# Eco-anxiety and ocean stewardship: evidence from a U.S. National Survey

**DOI:** 10.3389/fpsyg.2025.1680637

**Published:** 2025-11-27

**Authors:** Chris O’Halloran

**Affiliations:** Healthy Oceans, Healthy People, Santa Cruz, CA, United States

**Keywords:** eco-anxiety, eco-emotions, ocean conservation, pro-environmental behaviors, ocean stewardship

## Abstract

**Introduction:**

The present exploratory study investigates emotions, pro-environmental behaviors, and demographic predictors of ocean-related eco-anxiety.

**Methods:**

A nationally representative sample of 1,226 United States adults, standardized by age, gender, and geographic region, completed an online survey assessing psychological responses to ocean degradation, pro-environmental behaviors, and emotional connection to the marine environment.

**Results:**

The results of regression analysis revealed that individuals experiencing ocean-related eco-anxiety were more likely to report decreased depression near the ocean, express high concern about ocean conservation, reduce their carbon footprint, take action to protect the ocean, as well as hold the belief that ocean protection is the sole responsibility of the government. Furthermore, those who reported that fear motivates them to take ocean conservation action were also more likely to report experiencing high levels of eco-anxiety, suggesting that negative emotions can act as powerful drivers of ocean conservation action. With regard to age, among all age groups, adults aged 30–44 years old were significantly less likely to report ocean-related eco-anxiety.

**Conclusion:**

Taken together, these findings highlight the complex role of eco-anxiety in motivating conservation behavior and underscore the need to integrate emotional dimensions into future ocean stewardship efforts. Addressing eco-anxiety through conservation messaging and mental health interventions may strengthen emotional resilience and foster long-term commitment to ocean conservation.

## 1 Introduction

The global ocean covers approximately 71% of Earth’s surface and plays a central role in the worldwide biogeochemical processes of climate regulation, absorption of excess atmospheric heat, carbon cycling, oxygen production, and fresh water provision through the hydrological cycle (Intergovernmental Panel on Climate Change [IPCC], 2019, 2022). However, in the last several decades, the health of the world’s ocean has been threatened by numerous risks elicited by human activities, such as anthropogenic greenhouse gas emissions, which collectively caused sea-level rise, acidification, rising sea surface temperatures, deoxygenation, loss of marine biodiversity, and eco-anxiety/fear (Watts et al., 2017). Further harmful human activities include plastic pollution, overfishing, and destruction of coastal wetlands, all of which have resulted in the loss of marine biodiversity and habitat (UNESCO, 2021). As well as an environmental problem, marine degradation can also present as an emotional and cultural problem—particularly, for coastal communities whose livelihoods and identities are deeply rooted in the marine environment (UNESCO, 2021).

Climate change jeopardizes both planetary and human health. Many individuals develop eco-anxiety, broadly understood as the increased awareness about ecological concerns. The term “eco-anxiety” coined by Albrecht (2011), (p.43) initially referred to “a chronic fear of environmental doom.” To date, various definitions of eco-anxiety are used to address the challenging emotional responses to the existential threats posed of climate change and environmental degradation (UNESCO, 2021). While eco-anxiety does not constitute a medical diagnosis to be included in the Diagnostic Statistical Manual 5 of Mental Disorders (American Psychiatric Association, 2022), it can lead to or exacerbate mental health problems (Boluda-Verdú et al., 2022). For instance, anthropogenic climate change resulting in severe weather conditions (e.g., hurricanes, floods, wildfires, heat waves, droughts) can accelerate the development of mental health concerns such as acute anxiety, anger, shock, grief, substance abuse, depression, and post-traumatic stress disorder (Boluda-Verdú et al., 2022). Notably, Greta Thunberg’s eco-anxiety due to grief at governmental inaction led to weight loss, selective mutism, and depression. Greta’s eco-anxiety led to social activism through school strikes for climate action in front of the Swedish Parliament (Thunberg, 2018). Recognizing the impact of climate change on individuals and communities, in 2017, the American Psychological Association recommended empowering individual action through community support (American Psychological Association, 2017).

In the current literature, eco-anxiety is understood as a form of chronic or acute anxiety arising from awareness of environmental degradation and climate change (Clayton, 2020; Reyes et al., 2023) or as an emotional response to climate disruption (Intergovernmental Panel on Climate Change [IPCC], 2022). Encompassing both general worry about environmental decline (“eco-worry”) and more intense psychological symptoms such as panic attacks, insomnia, hopelessness, and obsessive thoughts (Clayton and Karazsia, 2020; Hickman et al.’s, 2021; Hogg et al., 2021), eco-anxiety is frequently experienced along with eco-grief, eco-depression, and eco-anger, all of which capture the emotional toll of living through the so-called Anthropocene, a period of significant and frequently irreversible human impact on the Earth’s ecosystems (Albrecht, 2011). Furthermore, as argued by Chalquist (2021), eco-anxiety would be more appropriately labeled “eco-fear,” as the corresponding feeling captures a normal and adaptive response to the existential threat of climate change. Furthermore, Kurth and Pihkala (2022) noted that “practical eco-anxiety” can be a beneficial emotional response to the ecological threats of climate change that, in the long run, can promote individual and planetary wellbeing. Importantly, available evidence suggests that eco-anxiety disproportionately affects those who feel closely connected to nature, youth, and coastal Indigenous communities that rely on the marine environment for their livelihoods (Clayton et al., 2014; Sanson et al., 2019; Hickman et al.’s, 2021; UNESCO, 2021). Furthermore, recognizing mental health impacts of the climate crisis, the World Health Organization [WHO] (2023), in the recommendation for the United Nations Decade of Ocean Science for Sustainable Development (2021–2030), proposed that the development goals include emotions to foster conservation engagement and reduce inaction due to feelings of overwhelm due to the dire state of the vast global ocean (see also Clayton et al., 2014; Intergovernmental Panel on Climate Change [IPCC], 2022).

According to recent estimates, incidence rates of eco-anxiety are currently growing. For instance, Hickman et al.’s (2021) found that over half of the surveyed respondents reported feeling sad, angry, or powerlessness about climate inaction. Other environmental studies found that approximately 30% of participants reported feeling overwhelmed by ocean degradation and the thought about what ocean conservation actions need to be taken (Stanley et al., 2021; Reyes et al., 2023). Furthermore, a recent IPCC report documented a strong association between emotional distress and increased public awareness of the environmental crises (Intergovernmental Panel on Climate Change [IPCC], 2022). Despite the fact that, to date, there is no agreed upon measurement tool for eco-anxiety or climate anxiety, recent validated screening tools to measure eco-anxiety include the 13-item Hogg Eco-Anxiety Scale, the 13-question the Climate Change Anxiety Scale, and the five-question Eco-Worry Scale (Hogg et al., 2021; Clayton and Karazsia, 2020; Verplanken et al., 2020; Parmentier et al., 2024). These eco-anxiety scales measure various eco-emotions (e.g., worry, anxiety, concern, overwhelm, fear, depression) that, depending on their severity, can either motivate conservation action (worry) or lead to the debilitating state of feeling overwhelmed and thus incapable of action (severe anxiety) (Clayton and Karazsia, 2020; Verplanken et al., 2020). In support of this assumption, Stanley et al. (2021) established that the feelings of hopelessness and/or being overwhelmed both hindered the survey participants’ conservation engagement and, alternatively, elicited eco-anger that later served as a motivator for personal behavior change.

In this context, this pilot study explored the association between eco-anxiety, defined as the feeling of being overwhelmed, and ocean conservation actions. The aim was to determine whether ocean-related eco-anxiety (binary: yes/no) was associated with demographic, emotions, well-being, awareness of ocean literacy principles, and ocean conservation behaviors. Specifically, the objectives of this exploratory research (a) estimated the prevalence of eco-anxiety among a nationally representative sample of American adults, (b) evaluated the association of eco-anxiety and self-reported ocean conservation actions and identified demographic, emotional and emotional wellbeing associations and (c) evaluated the association between ocean related eco-anxiety and self-reported conservation engagement. The study null hypothesis stated there was no association between eco-anxiety and ocean conservation engagement. This study specified two alternative hypotheses for the eco-anxiety and ocean conservation relationship: eco-anxiety was associated with no ocean conservation engagement, and eco-anxiety motivated engagement in ocean conservation. The study also tested the following directional hypotheses: (1) respondents who were “very concerned” about the ocean would be more likely to engage in conservation; (2) stronger reliance on the government to protect the ocean would be associated with higher eco-anxiety; and (3) younger respondents would report higher eco-anxiety. In summary, this pilot study explored the association between eco-anxiety and ocean-conservation engagement.

## 2 Materials and methods

### 2.1 Participants

The data were collected using a cross-sectional anonymous online survey of U.S. adults standardized for gender, age, and geographic region. The survey was conducted on 18–19 May 2022. Eligibility criteria included: (1) being 18 years or older, (2) residence in the United States, (3) literacy in English, and (4) Internet access to complete the survey. The participants were recruited through Survey Monkey/Momentum which provides global market research. Millions of people volunteer to complete Survey Monkey survey’s in order to win a chance for a sweepstakes, earn credit for gift cards, or donate money to charity. Survey respondents share demographic information in order to target desired populations. A total of 1,302 American adults began the survey, however, 62 people (4.8%) did not consent to participate and the survey ended. An additional 14 individuals consented to participate but did not proceed to take the survey. The overall survey response was 94%. A total of 1,226 eligible respondents completed the questionnaire and provided informed consent. All responses were self-reported. Questions were single answer or multiple choices. On average it took participants 5 min to complete the survey.

### 2.2 Ethical approval

The study protocol was reviewed and approved by the Institutional Review Board of Ethical and Independent Review Services (Protocol #22097–01), now known as Salus IRB. Informed consent was obtained electronically prior to participation, and individuals who did not consent were unable to proceed with the survey.

### 2.3 Instruments

The online questionnaire, administered using Momentum^1^, was comprised of closed-ended questions. Ocean-related eco-anxiety was assessed using a single item: “I am overwhelmed by the dire state of the ocean and what I can do to help.” This single item exploratory measure captures the affective dimension of eco-anxiety, reflecting emotional distress and feelings of being overwhelmed by environmental degradation (Clayton and Karazsia, 2020; Pihkala, 2020). The statement uses both scientific and public discourse about ocean decline, thereby enhancing its ecological validity and resonance with real-world ecological concerns. This single-item question is not as comprehensive of a measure as the multidimensional structure of eco-anxiety as defined in validated instruments such as the Hogg Eco-Anxiety Scale (HEAS). HEAS assesses four sub-dimensions: affective symptoms, rumination, behavioral symptoms, and anxiety about one’s personal environmental impact (Hogg et al., 2021). Consequently, while this item effectively represents emotional overwhelm, it may underrepresent cognitive and behavioral aspects of eco-anxiety.

In terms of demographic information, the participants provided details such as ethnicity, age, gender, region they lived in, income level, and education level by selecting one choice of a multiple option question. The survey also collected data on:

- Distance lived from the ocean (six categories),
- Frequency of annual beach visits (eight categories)
- Activities > 12 in the past year - multiple option checklist (e.g., ocean activities; beach activities; walking/running/hiking; gym; biking; racquet sports)
- Awareness of ocean literacy principles, nine multiple options checklist (e.g., “The ocean supports a great diversity of life and ecosystems”; “The ocean is a major influence on weather and climate”)
- Very concerned about ocean conservation (yes/no)
- Emotions influencing ocean conservation engagement - multiple options (e.g., joy, awe, love, interest/wonder, fear, anger, sadness, contentment…)
- Perceived impact of the ocean on health - multiple option checklist - (e.g., calming/less stress; increased happiness; emotional wellbeing; decreased depression; spiritual refuge)
- Influence on ocean and beach behaviors in the past year (e.g., nature documentaries, books, social media, campaigns, aquariums, hands on experiences, classes, none…)
- Ocean benefits, and actions responsibilities (e.g., it’s the sole responsibility of government to protect the ocean, individual actions can have a positive impact on ocean conservation…)
- Engagement with ocean conservation issues (e.g., participated in beach cleanup, worked or volunteered for a marine conservation organization, donated money to ocean non-profits…)

### 2.4 Data analysis

The required sample size was determined using the data from the 2020 Census Bureau of American adults (258,300,000). The results revealed that a total of 1,067 participants would be needed to obtain a confidence level of 95% and a confidence interval of 3%. Accordingly, a total of 1,226 eligible respondents were included in the final sample.

All analyses were performed using Stata 15 (StataCorp LP, College Station, TX, United States). Descriptive statistics summarized participant characteristics. Bivariate analyses were conducted to examine associations between eco-anxiety and potential predictors, including gender, age, education, ethnicity, annual ocean visits, frequency of ocean and beach activities, ocean literacy awareness, emotional benefits or risks associated with ocean exposure, ocean conservation actions, and swimming ability. Bivariate associations were explored with Pearson correlations among binary covariates (*p* < 0.05).

Variables with *p* < 0.05 in bivariate analyses were included in a multivariable logistic regression model estimating associations between the dichotomous outcome variable (eco-anxiety) and key explanatory variables. Covariates included age, fear as a motivator for ocean conservation, engagement in conservation actions, reduced carbon footprint, perceptions of government responsibility for ocean protection, decreased depression due to ocean exposure, and concern for ocean conservation. Adjusted risk ratios (RR) and 95% confidence intervals were calculated for all predictors.

### 2.5 Research design

This study employed a cross-sectional quantitative design to explore the relationship between ocean-related eco-anxiety and demographic, behavioral, and emotional predictors of pro-environmental engagement. The design was intended to identify associations rather than infer causality, providing foundational evidence for future more rigorous longitudinal and experimental research examining eco-anxiety and ocean conservation behavior.

## 3 Results

The survey participants’ (*N* = 1,226) demographic characteristics are summarized in Table 1. Gender, age, and location were standardized with U.S. census data for the American adult population. Most of the study participants were White (69%), female (51.8%), between 45 and 60 years old (28%), and earned less than $50,000 annually (34%). Twenty-seven percent of the study cohort (328) reported feeling overwhelmed by the dire state of the ocean and what they could do to help.

**TABLE 1:** Characteristics of the study participants (*N* = 1,226).

Characteristics	*N*	%
**Age (years)**		
18–29	263	21.47
30–44	311	25.39
45–60	346	28.24
>60	305	24.90
**Gender**		
Female	634	51.76
Male	591	48.24
**Ethnicity**		
Asian	153	12.48
African American/Black	114	9.30
American Indian/Alaska Native	32	2.61
White	849	69.25
Latino	149	12.15
**Education**		
Graduated 4-year college	268	21.86
Did not graduate from 4-year college	957	78.14
Household income (annual)		
<$50,000	421	34.37
Between $50,000 and 100,000	375	30.62
>$100,000/year	305	24.89
Prefer not to answer	124	10.12

The results of the logistic regression analysis revealed the associations among demographic characteristics, ocean conservation attitudes and behaviors, and the likelihood of experiencing eco-anxiety related to the ocean. The dependent variable was binary (1 = experiences ocean-related eco-anxiety; 0 = does not). A total of seven variables were found to be significant predictors of eco-anxiety—namely, very concerned about ocean conservation, fear motivating ocean conservation action, belief that it is the governments sole responsibility to protect the ocean, decreased depression near the ocean, reduced carbon footprint, age, and engaged in actions to protect the ocean environment (see Table 2).

**TABLE 2:** Logistic regression predicting ocean-related eco-anxiety (*N* = 1,226).

Variable	RR	*P*	95% CI
Age 30–44	0.924	0.001	[0.881, 0.968]
Fear motivates me to take action for conservation	1.202	<0.001	[1.132, 1.277]
Reduced my carbon footprint	1.111	<0.001	[1.059, 1.165]
I take actions to protect the ocean & marine environment	1.077	0.002	[1.028, 1.128]
It’s the sole responsibility of government to protect the ocean	1.131	0.001	[1.052, 1.216]
I am very concerned about ocean conservation	1.301	<0.001	[1.243, 1.362]
Decreased depression being near the ocean	1.124	<0.001	[1.072, 1.179]

RR, risk ratio; CI, confidence interval. Values are based on logistic regression with robust standard errors. The dependent variable is a binary indicator of ocean-related eco-anxiety (1, yes; 0, no).

Accordingly, these seven independent variables were added to the final model (see Table 2) to be tested using a multivariable logistic regression analysis. All predictors except the constant were statistically significant at *p* < 0.01. The results revealed that the respondents who reported feeling very concerned about ocean conservation were 30% more likely to report ocean-related eco-anxiety (RR = 1.30, 95% CI [1.24, 1.36], *p* < 0.001). This indicates that heightened emotional concern aligns with greater ocean conservation engagement.

Furthermore, the participants who agreed that fear motivated them to take action for conservation had a 20% increased likelihood of experiencing ocean-related eco-anxiety (RR = 1.20, 95% CI [1.13, 1.28], *p* < 0.001), suggesting that negative emotions may serve as a motivator. Similarly, the individuals who reported having reduced their carbon footprint (RR = 1.11, *p* < 0.001), taken action to protect the ocean (RR = 1.08, *p* = 0.002), or felt decreased depression near the ocean (RR = 1.12, *p* < 0.001) also had a significantly higher probability of reporting ocean-related eco-anxiety. This suggests that individuals who engage in conservation behaviors experience stronger eco-emotional responses.

The belief that ocean protection is the sole responsibility of the government was associated with a 13% increase in the likelihood of experiencing eco-anxiety (RR = 1.13, 95% CI [1.05, 1.22], *p* = 0.001). This suggests that reduced personal agency increases feelings of distress when facing environmental protection. Finally, the individuals aged 30–44 years old were less likely to experience ocean-related eco-anxiety, with a 7.6% reduction in likelihood (RR = 0.92, 95% CI [0.88, 0.97], *p* = 0.001). This suggests a lower emotional engagement among this cohort.

Table 3 summarizes the means and standard deviations (SD) for covariates that were not statistically significant in the final regression model. Table 4 shows that after adjustment in the logistic regression, none of the following variables were associated with eco-anxiety (*p* > 0.05): >12 ocean activities/year, >12 beach activities/year, not supporting ocean conservation, encouraging others to support conservation, participating in beach cleanups, working/volunteering for an environmental organization, donating to ocean non-profits, eating sustainable seafood, voting for ocean conservation, reducing plastic use, recycling, living less than 1 miles to the ocean or living between 1 and 10 miles to the ocean. Several variables showed small bivariate correlations with eco-anxiety, but these associations did not persist after adjustment in the logistic model, indicating they do not explain variance with eco-anxiety beyond other covariates.

**TABLE 3:** Variables, means, and standard deviations (SD) for covariates not significant in the final regression model (*N* = 1,226).

Variable	Mean	SD
Eco-anxiety	0.255	0.436
>12 ocean activities/year	0.200	0.401
>12 beach activities/year	0.233	0.423
Didn’t support ocean conservation	0.186	0.390
Encouraged others to support ocean conservation	0.279	0.449
Beach cleanup	0.144	0.351
Worked/volunteered for environmental org	0.073	0.260
Donated to ocean nonprofit	0.141	0.349
Ate sustainable seafood	0.238	0.426
Voted for ocean conservation	0.152	0.359
Reduced plastic use	0.375	0.484
Recycled	0.459	0.499
Live < 1 mile from ocean	0.035	0.184
Live 1–10 miles from ocean	0.151	0.358

SD, standard deviation.

**TABLE 4:** Intercorrelations with eco-anxiety for covariates non-significant in the final regression model (*N* = 1,226).

	1	2	3	4	5	6	7	8	9	10	11	12	13	14
1. Eco-anxiety	1.000	–	–	–	–	–	–	–	–	–	–	–	–	–
2. >12 ocean activities/year	0.0501	1.000	–	–	–	–	–	–	–	–	–	–	–	–
3. >12 beach activities/year	0.0993*	0.4768*	1.000	–	–	–	–	–	–	–	–	–	–	–
4. Didn’t support ocean conservation	−0.0877*	−0.1002*	−0.1366*	1.000	–	–	–	–	–	–	–	–	–	–
5. Encouraged others to support ocean conservation	0.2087*	0.2079*	0.2021*	−0.2622*	1.000	–	–	–	–	–	–	–	–	–
6. Beach cleanup	0.0247	0.2208*	0.2089*	−0.1564*	0.1402*	1.000	–	–	–	–	–	–	–	–
7. Worked/volunteered for environmental org	0.0688*	0.1205*	0.1207*	−0.1267*	0.1384*	0.2084*	1.000	–	–	–	–	–	–	–
8. Donated to ocean nonprofit	0.1771*	0.1198*	0.1613*	−0.1714*	0.1951*	0.1388*	0.1517*	1.000	–	–	–	–	–	–
9. Ate sustainable seafood	0.1466*	0.117*	0.1238*	−0.2252*	0.1166*	0.0105	0.0396	0.1348*	1.000	–	–	–	–	–
10. Voted for ocean conservation	0.1987*	0.1173*	0.1397*	−0.1752*	0.2137*	0.1469*	0.1636*	0.2686*	0.2001*	1.000	–	–	–	–
11. Reduced plastic use	0.2658*	0.1258*	0.2341*	−0.3293*	0.2096*	0.0033	0.0604*	0.1098*	0.2919*	0.2439*	1.000	–	–	–
12. Recycled	0.2625*	0.0877*	0.2148*	−0.3851*	0.15*	−0.0665*	−0.013	0.1227*	0.2874*	0.1996*	0.5466*	1.000	–	–
13. Live < 1 mile from ocean	0.0052	0.0949*	0.1052*	−0.0477	0.0419	0.0908*	0.0766*	0.0806*	0.0129	0.0488	0.0275	0.0708*	1.000	–
14. Live 1–10 miles from ocean	−0.0022	0.1091*	0.117*	−0.0456	0.0721*	0.1183*	0.0487	0.016	0.0453	0.1236*	0.0105	−0.0309	−0.0447	1.000

*Indicates *p* < 0.05. Correlations are Pearson’s r (two-tailed). Variable list: 1, Eco-anxiety; 2, >12 ocean activities/year; 3, >12 beach activities/year; 4, didn’t support ocean conservation; 5, encouraged others to support ocean conservation; 6, beach cleanup; 7, worked/volunteered for environmental org; 8, donated to ocean nonprofit; 9, ate sustainable seafood; 10, voted for ocean conservation; 11, reduced plastic use; 12, recycled; 13, Live < 1 mile from ocean; 14, Live 1–10 miles from ocean.

## 4 Discussion

The pilot study findings revealed an association between respondents’ concern for ocean conservation and ocean-related eco-anxiety, indicating the complex emotions elicited by environmental concern. Overall, several meaningful patterns can be discerned in the results. First, eco-anxiety was found to be 30% more prevalent among individuals who reported being very concerned about ocean conservation. More specifically, the survey participants who reported taking concrete pro-environmental actions such as reducing their carbon footprint or participating in efforts to protect marine environments were more likely to experience ocean-related eco-anxiety. Similarly, the participants who acknowledged decreased depression when spending time near or in the ocean also reported higher rates of eco-anxiety. These findings align with several previous studies that identified an association between eco-anxiety and environmental action (Clayton, 2020; Stanley et al., 2021), suggesting that heightened environmental concern may serve as a psychological driver of action rather than just a symptom of distress.

Second, the survey respondents who admitted experiencing fear as a catalyst for conservation actions were 20% more likely to experience ocean-related eco-anxiety. This finding supports Chalquist (2021) previously described the concept of *blue fear*, an emotionally rational response to environmental degradation, which can lead to conservation action. This is consistent with Stanley et al. (2021) findings that while eco-anger and eco-anxiety were associated with increased environmental activism, eco-depression did not induce such effect. Instead, eco-depression was linked to reduced environmental action and decreased wellbeing (Stanley et al., 2021). In this context, our finding that fear can serve as a strong motivator for pro-environmental behavior, rather than lead to inaction, suggests that, unlike depression, fear is cognitively appraised as actionable and can spur active behavioral engagement in ocean conservation, similarly to how eco-anger may channel frustration into activism. Additionally, this finding also highlights the complexity of the emotional landscape of concern for ocean conservation, with different emotions and affective states (fear, anger, frustration, depression) eliciting varying responses that, in turn, drive different forms of behavioral (dis)engagement.

Third, the belief by participants that ocean protection is the sole responsibility of the government was associated with a 13% higher likelihood of experiencing eco-anxiety (RR = 1.13). This outcome may suggest that those respondents who believed it was the responsibility of government to address large global problems feel a greater sense of helplessness when faced global environmental problems. This interpretation is consistent with the concept of eco-anger, in which frustration at systemic inaction contributes to emotional distress and can also led to a desire to advocate for change (Stanley et al., 2021; Hogg et al., 2021). Similarly, Hickman et al.’s (2021) survey of global youth found that government inaction increased emotional distress, eco-anxiety, and frustration about institutional inaction, all of which can drive local, community-based, and small individual actions within larger-scale ocean conservation campaigns and initiatives.

The fourth pattern identified in the data highlights that the respondents who reported experiencing decreased depression from spending time in or near the ocean were more likely to report eco-anxiety. This finding corroborates previous research on place attachment and the so-called solastalgia (Albrecht et al., 2007), broadly understood as one’s feelings of grief, dread, and distress elicited by observing degradation or destruction of a place that provides one with healing, identity, or meaning, This emotion, coupled with the realization that the ocean is both a life-sustaining ecological system and a place of emotional and physical wellbeing may heighten emotional sensitivity to its degradation, and may intensifying emotional investment in ocean stewardship.

Finally, the participants aged 30–44 years were less likely to report ocean-related eco-anxiety, showing a 7.6% reduction in likelihood (RR = 0.92). This finding contradicts the results of several previous studies showing that younger adults report more concern about climate change and more actively engage in climate change activism (Reyes et al., 2023). One tentative interpretation may be that individuals in this age group face greater employment and/or family obligations which may limit their engagement in ocean stewardship. Therefore, what these findings also suggest is that ocean conservation initiatives and campaign messages may need to address different age groups and lifestyle concerns. Similarly, mental health counselors could frame eco-anxiety as a normal response to environmental degradation and loss of biodiversity, rather than a pathological issue. Counselors could help people to make the small positive actions they want to make to address environmental change and transform being overwhelmed, anxious, and distressed into resilience and ocean stewardship. However, it should also be noted that, if people feel overwhelmed and not supported, chronic eco-anxiety can lead to burnout and disengagement. While eco-anxiety is a strong driver of environmental action, emotional support and community-based tools are also important in terms of addressing the existential threat and anxiety of environmental degradation (Clayton et al., 2014; World Health Organization [WHO], 2023).

Within the framework of Protection Motivation Theory (PMT), perceptions of threat and coping efficacy determine whether fear-based emotions such as eco-anxiety lead to adaptive or maladaptive outcomes. Knowledge and understanding are core components of environmental and ocean literacy and play an important role in shaping these cognitive appraisals. Individuals with higher levels of ocean literacy are more likely to interpret environmental threats as actionable rather than overwhelming. Conversely, individuals with limited understanding of ocean ecosystems or uncertainty about effective solutions may have increased anxiety and feelings of helplessness. Integrating educational and emotional aspects within the PMT framework demonstrates how increasing ocean literacy can serve as an emotional buffer to transform fear and overwhelm into environmental action.

In addition, ocean literacy, defined as an understanding of the ocean’s influence on humans and humans’ influence on the ocean, provides an important framework to understand cognitive and affective aspects of pro-environmental behavior (Cava et al., 2005). Additionally, ocean literacy encompasses knowledge, communication, and responsible decision-making about ocean ecosystems (Fauville et al., 2019). Research studies have found that higher levels of ocean literacy are associated with greater emotional engagement with ocean stewardship (Chen and Tsai, 2016; O’Halloran, 2025). Given that eco-anxiety, eco-anger, and eco-depression represent affective responses to ecological degradation, future research should explore how ocean literacy impacts these emotions in shaping ocean conservation behavior. Ongoing global initiatives to enhance ocean literacy (Kao et al., 2025; Choi et al., 2024) provide an opportunity to examine how ocean stewardship campaigns can build emotional resilience, strengthen collective efficacy, and foster coping in the face of increasing ocean threats.

Finally, future research should explore how community-level variables such as social norms, collective efficacy, and cultural identity may mediate the link between eco-anxiety and ocean conservation behavior. Buchan’s (2021) research on *marine citizenship*—a socially and psychologically grounded sense of responsibility and participation in ocean stewardship—requires recognizing the community and cultural contexts that shape individuals’ environmental engagement. Given that ocean ecosystems hold cultural, spiritual, and identity value for many coastal and Indigenous communities, understanding these relational dimensions may help to develop inclusive, emotionally informed ocean conservation strategies.

### 4.1 Limitations and future implications

The present pilot study had several strengths and limitations. First, although the study analyzed a large cohort of U.S. adults standardized for gender, age, and location, the observational design of the present study may have led to unmeasured confounding. Second, considering that all data used for the analyses were self-reported, we cannot rule out the risks of information bias, recall bias, or social desirability bias. A third relevant concern is selection bias, as the recruited participants, individuals who explicitly volunteered to complete the survey, may have different characteristics than those who chose not to participate. An additional source of bias is that online surveys may exclude or underrepresent populations with limited or no access to the Internet. Another potential limitation of the present cross-sectional research design is that it does not allow one to unambiguously establish causal relationships, as data were collected at a single point in time.

To address the limitations of this exploratory study, future research would benefit from the use of validated measurements, such as the Hogg Eco-Anxiety Scale (Hogg et al., 2021) or climate worry scales (Stewart, 2021). Because single-item measures probably do not capture the multidimensional nature of eco-anxiety, future research should employ validated multi-item scales to assess emotional, cognitive, and behavioral aspects more comprehensively (Hogg et al., 2021). Additionally, all constructs including eco-anxiety and all covariates were measured with single-item questions. Reliance on single-item measures may limit reliability, underrepresent the multidimensional nature of the constructs assessed, and bias observed associations. In addition, longitudinal or experimental designs would allow stronger causal inferences (Lange and Dewitte, 2019). Finally, given that the present pilot study focused on a representative sample of American adults, future research involving international and Island Nation cohorts would be valuable to determine whether the identified associations hold across diverse populations and cultural contexts.

## 5 Conclusion

Eco-anxiety, an emotional response to anthropogenic climate change, comprises an array of ecology-related feelings. In the context of the current climate change crisis and the associated ocean ecological crisis, it is essential to understand individuals’ responses to ecology-related issues that may catalyze ocean environmental stewardship and systemic change. Accordingly, the present study sought to evaluate ocean-related eco-anxiety in a representative sample of U.S. adults. The results revealed that eco-anxiety is a motivator of ocean conservation action. Specifically, the findings of this pilot study showed that eco-anxiety, specifically the feeling of being overwhelmed by the dire state of the ocean environment, was positively associated with ocean conservation engagement. The results highlight that incorporating ecological emotions in ocean conservation campaigns/initiatives and mental health interventions can effectively foster emotional resilience, enhance ocean stewardship, and promote individual health and wellbeing.

## Data Availability

The data supporting the conclusions of this article can be made available on request. Requests to access the dataset can be directed to the corresponding author.

## Footnotes

1
https://www.surveymonkey.com/
